# Effects of 10-week walking and walking with home-based resistance training on muscle quality, muscle size, and physical functional tests in healthy older individuals

**DOI:** 10.1186/s11556-018-0201-2

**Published:** 2018-11-19

**Authors:** Akito Yoshiko, Aya Tomita, Ryosuke Ando, Madoka Ogawa, Shohei Kondo, Akira Saito, Noriko I. Tanaka, Teruhiko Koike, Yoshiharu Oshida, Hiroshi Akima

**Affiliations:** 1 Graduate School of Medicine, Nagoya University, Nagoya, Japan; 2 Graduate School of Education and Human Development, Nagoya University, Nagoya, Japan; 3 Japan Society for the Promotion of Science, Tokyo, Japan; 4 Research Center of Health, Physical Fitness and Sports, Nagoya University, Nagoya, Japan; 5 Faculty of Sports Sciences, Waseda University, Saitama, Japan; 6 School of International Liberal Studies, Chukyo University, Toyota, Japan

**Keywords:** Echo intensity, Home-based resistance training, Muscle quality, Walking, Ultrasonography

## Abstract

**Background:**

Older individuals have been shown to present muscle atrophy in conjunction with increased fat fraction in some muscles. The proportion of fat and connective tissue within the skeletal muscle can be estimated from axial B-mode ultrasound images using echo intensity (EI). EI was used to calculate the index of muscle quality. Walking, home-based weight-bearing resistance training, and its combinations are considered simple, easy, and practical exercise interventions for older adults. The purpose of this study was to quantify the effects of walking and walking with home-based resistance training on muscle quality of older individuals.

**Methods:**

Thirty-one participants performed walking training only (W-group; 72 ± 5 years) and 33 participants performed walking and home-based resistance training (WR-group; 73 ± 6 years). This study was a non-randomized controlled trial with no control group. All participants were instructed to walk 2 or 3 sets per week for 10 weeks (one set: 30-min continuous walking). In addition, the WR-group performed home-based weight-bearing resistance training. EI was measured as a muscle quality index using axial B-mode ultrasound images of the rectus femoris and vastus lateralis of the mid-thigh. We further averaged these parameters to obtain the EI of the quadriceps femoris (QF). Participants further performed five functional tests: sit-ups, supine up, sit-to-stand, 5-m maximal walk, and 6-min walk.

**Results:**

QF EI was significantly decreased in both groups after training (W-group 69.9 ± 7.4 a.u. to 61.7 ± 7.0 a.u., WR-group 64.0 ± 9.5 a.u. to 51.1 ± 10.0 a.u.; *P* < 0.05), suggesting improved muscle quality. QF EI was further decreased in the WR-group compared with the W-group. The sit-up test in both groups and the sit-to-stand and 5-m maximal walk tests in the W-group were significantly improved after training.

**Conclusion:**

These results suggest that training-induced stimulation is associated with a decrease in EI in some thigh regions. Furthermore, the addition of home-based resistance training to walking would be effective for a greater reduction of EI.

## Background

Skeletal muscle mass and function decline with age, and this age-related deterioration of skeletal muscle is known as sarcopenia [1]. As a result of aging and the progression of sarcopenia, adipose tissue infiltrates the skeletal muscle. Increased fat infiltration within the muscle, i.e., increased intramuscular fat (IMF) content, which can be assessed by computed tomography (CT) and magnetic resonance imaging (MRI), is considered to reflect worse muscle quality [2–4]. Aging-related physical and metabolic impairments have been commonly investigated in many previous studies [5, 6]; however, little attention has been paid to the role that IMF may play in these processes. Further, excessive IMF was found to be related to lower maximum strength, lower gait ability, and insulin resistance [7–9]. These findings imply that worse muscle quality may cause difficulty in living independently as well as metabolic syndrome. Thus, practical and effective methods of improving muscle quality as well as decreasing IMF in older individuals are needed.

Exercise interventions utilizing endurance and resistance training protocols based on maximal oxygen consumption and one-repetition maximum, respectively, have been reported to induce significant muscle hypertrophy and improvements in cardiovascular function that enhance overall physical function in older adults [10, 11]. Besides these interventions, there are few attempts at determining whether physical training reduces the IMF content in middle-aged and older individuals [12]. Walking and home-based weight-bearing resistance training have been proposed as simple, easy, and practical exercise interventions for older adults. Walking in particular has been shown to effectively improve physical function [10, 13] and insulin responsiveness [14] and to reduce abdominal fat [15]. Previously, Ryan et al. [16] evaluated the effects of walking combined with diet restriction on IMF cross-sectional area in older obese women using CT. They showed a decrease in IMF cross-sectional area after 6 months of the intervention. Thus, in this study, we hypothesize that an increase in physical activity (i.e., walking) would also improve muscle quality; however, the effects of walking training in older individuals on muscle quality are not well understood.

A combination of traditional endurance and resistance training has also been shown to improve muscle quality in older individuals. Wilhelm et al. [17] showed that concurrent strength and endurance training increased muscle thickness and decreased ultrasound echo intensity (EI), which reflects IMF and/or connective tissue content. Higher skeletal muscle EI is associated with lower muscle quality [18, 19]. Previous studies have shown that a statistically significant correlation exists between muscle EI and adipose tissue level in a muscle biopsy sample [20]. Furthermore, connective and fibrous tissue also reflect muscle EI [21, 22]. Akima et al. [23] showed significant correlation between EI and extramyocellular lipid levels determined by ^1^H magnetic resonance spectroscopy (MRS) and IMF content measured using MRI. Therefore, EI can potentially reflect the lipid content around muscle cells; however, it should be paid a little attention to including connective and fibrous tissue as well. These observations suggest that combined training can improve muscle quality by reducing the IMF content. Additionally, in a cross-sectional study, Akima et al. [3] found that IMF content measured by MRI was related to muscle size (*r* = − 0.67 to − 0.59, *P* < 0.05), suggesting that individuals with larger muscles have less IMF. Considering the results of these two studies, it appears that an increase in muscle size is the key to IMF reduction and that a combination of walking and resistance training would effectively reduce IMF; however, this has not been proven. Home-based weight-bearing resistance training is currently being recommended for sarcopenic individuals because this type of training has been shown to improve not only strength and functional ability but also muscle size [13, 24, 25]. Accordingly, we hypothesize that the combination of walking and home-based weight-bearing resistance training may decrease IMF and eventually improve muscle quality, more than walking training alone. The purpose of this study was to compare the effects of walking alone and walking combined with home-based weight-bearing resistance training on the muscle quality of the thigh muscles of older individuals.

## Methods

### Experimental design and procedure

This study was carried out as part of health promotion classes for volunteers at Nagoya City from 2014 to 2015. All participants learned about this class through a public relations magazine or website, and they applied to participate in the class on their own accord. The class consisted of an introductory session (1st week), measurement sessions (2nd and 12th week), lectures on health promotion (4th, 6th, 8th, and 10th week), and presentation of the measurement results (13th week). Therefore, we met the participants at least once every 2 weeks during the class. At the first visit, we introduced the purpose and significance of the study to the participants and explained the entire experimental protocol and specific training procedures and measurement techniques.

All participants gave their written informed consent before study participation. Participants were assigned non-randomly to two exercise groups (i.e., walking alone or walking combined with resistance training) and performed the prescribed exercises at home for 10 weeks; they recorded their training on customized recording sheets. This study was designed as a non-randomized controlled trial to unify the training condition within the class. Practical considerations required that all participants in each class perform the same exercise; therefore, participants were assigned to the walking group (W-group) during the first year (2014) and to the walking combined with resistance training group (WR-group) during the second year (2015). Nobody attended the class during both the first and second years. During the study period, the participants were instructed to avoid changes in diet and in their recreational physical activities (e.g., walking, jogging, and stretching). Before and after the training intervention, the participants underwent skeletal muscle ultrasonography and physical function testing in our laboratory.

### Participants

The participant inclusion criteria were as follows: 1) resided in Nagoya City, 2) aged 65 years or older, 3) did not have any conditions requiring exercise restriction (e.g., cardiac disease, respiratory disease, hypertension, orthopedic conditions), 4) were able to perform activities of daily living (ADL) independently, and 5) were not currently involved in exercise training. Questionnaires and interviews during the first classroom session were used to determine pre-existing conditions and the ability to perform ADL, and no participants were excluded based on these issues. Seventy-nine healthy older men and women were eligible to participate in this study. Fifteen participants failed to complete either the 10-week intervention or the follow-up examination; however, none of these participants dropped out because of injury or illness. Of the 64 participants who completed the study, 31 were assigned to the W-group (16 men and 15 women; age 72 ± 5 years, height 159 ± 8 cm, weight 56 ± 10 kg, BMI 22 ± 3 kg/m^2^) and 33 were assigned to the WR-group (12 men and 21 women, age 73 ± 6 years, height 156 ± 7 cm, weight 53 ± 7 kg, BMI 22 ± 2 kg/m^2^). Before the experiment, the purpose, procedures, and risks associated with this study were explained to each participant, and written informed consent was obtained from all participants. All examination protocols were approved by the Institutional Review Board of the Research Center for Health, Physical Fitness and Sports at Nagoya University (approval numbers: 26–13, 27–9) and conducted in accordance with the ethical principles stated in the Declaration of Helsinki.

### Training program

The walking program performed by W- and WR-group participants consisted of more than two sets per week. One set included at least 30 min of continuous walking without rest. Furthermore, participants were asked to try to achieve an average of 10,000 steps per day (i.e., total of 70,000 steps per week). If the daily steps of the participants exceeded 10,000 steps without the walking intervention, we instructed them to add the opportunity for walking more than two sets per week. In this case, they would not worry about the target step numbers. Each participant was instructed to walk at his/her usual speed. The participants wore a pedometer (PD-635, TANITA, Tokyo, Japan) attached to the anterior midline of their waist while performing their ADL every day during the 10-week training period from the time they got up in the morning until they went to bed at night. The reliability of this type of pedometer has been well established, and these pedometers are widely used to measure step counts [26, 27].

The WR-group performed resistance training at least three times per week during the 10-week training period. We used an original home-based resistance training program developed by the Japan Health Promotion & Fitness Foundation. This program can be performed at home and does not require any specialized exercise equipment. The participants were instructed on the correct exercise techniques during the first classroom session and were then able to perform the training at home while watching the DVD, which included a performance model with music and explanation of key points of the exercise. The training regimen consisted of five exercises: chair stands, hip flexions, calf raises, lateral leg raises, and sit-ups. Chair stands consisted of repeatedly standing and sitting on a chair. Hip flexions consisted of alternate elevations of each knee until the hip joint was flexed at a 90° angle. Calf raises consisted of alternating plantar flexion and dorsiflexion while standing. Lateral leg raises consisted of adduction of each leg to approximately 30° from the vertical while standing. Sit-ups were performed from a supine position, with the knees bent at approximately 80° and arms crossed in front of the chest. Hip flexions, calf raises, and lateral leg raises were performed in a standing position while holding a chair to support the body. Participants repeated 45 repetitions of each exercise to the music on the DVD and were told to perform these exercises while singing to the lyrics to avoid holding their breath. It was estimated that the participants would complete one series in approximately 30 min. Between each exercise, the participants were required to take a 30-s break. We instructed the participants to keep training logs using the customized recording sheets that we provided. We also required participants to record their physical condition, number of steps/day, type and frequency of physical activities, and special events in their daily life. We checked that the participants were properly completing the training tasks by interview once every 2 weeks.

### Ultrasound measurements

**Fig. 1:**
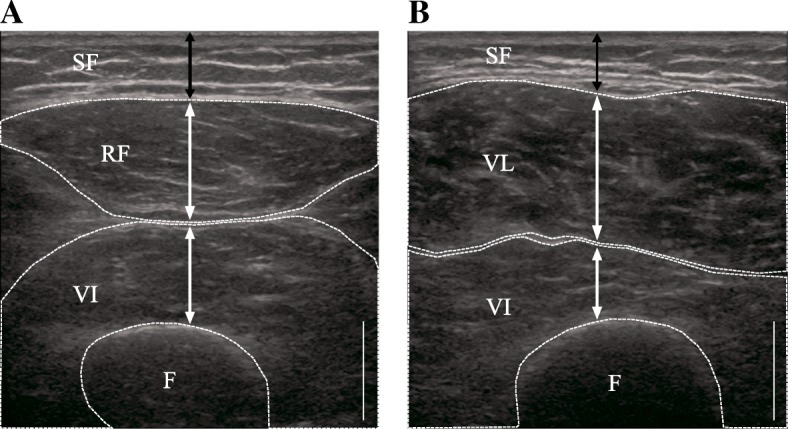
Representative ultrasound images of the anterior (**a**) and lateral (**b**) thighs. SF, subcutaneous fat; RF, rectus femoris; VI, vastus intermedius; VL, vastus lateralis; F, femur. Black double-headed arrows show subcutaneous fat thickness. White double-headed arrows show muscle thickness. Scale is 1 cm

Subcutaneous fat thickness, muscle thickness, and EI of the mid-thigh were measured by ultrasonography, as in our previous study [19, 28, 29]. Ultrasonography was performed after 15 min rest in order to avoid the influence by the body fluid shifts induced by muscle contraction [30]. Participants laid down on an examination bed in a supine position, with their knee joints fully extended. We measured the anterior and lateral parts of the right thigh corresponding to the midpoint between the greater trochanter and lateral condyle. A real-time B-mode ultrasonography device (LOGIQ e, GE Healthcare, Duluth, GA, USA) with a 3.8-cm, 8–10 MHz linear array probe was used to obtain images (Fig. 1) with the following acquisition parameters: frequency 10 MHz, gain 70 dB, depth 4.0 to 6.0 cm, focus point 1 (top of the image). The depth was determined depending on each participant, generally up to 6.0 cm, and was set at the same level before and after the training period. A water-soluble gel was applied to the scanning head of the probe to achieve acoustic coupling, and extra care was taken to avoid deformation of the muscle architecture. Three frozen axial images of each section were stored in the DICOM format and transferred to a personal computer. ImageJ software (National Institute of Health, Bethesda, MD, USA, version 1.46) was used for analysis. The thickness of subcutaneous fat was identified as the distance between the dermis and upper boundary of the ventral fascia. Muscle thickness (MT) in the rectus femoris (RF) and vastus lateralis (VL) was defined as the distance between the superior border of the subcutaneous fascia and the deep aponeurosis. In the case of the vastus intermedius (VI), muscle thickness was defined as the distance between the inferior border of the superficial aponeurosis and the superior border of the femur. The subcutaneous fat thickness (SFT QF) and muscle thickness (MT QF) of the quadriceps femoris (QF) were calculated using the following equations:

$$ \mathrm{SFT}\ \mathrm{QF}=\left(\mathrm{anterior}\ \mathrm{subcutaneous}\ \mathrm{fat}\ \mathrm{thickness}+\mathrm{lateral}\ \mathrm{subcutaneous}\ \mathrm{fat}\ \mathrm{thickness}\right)/2 $$

$$ \mathrm{MT}\ \mathrm{QF}=\left(\mathrm{thickness}\ \mathrm{of}\ \mathrm{RF}+\mathrm{thickness}\ \mathrm{of}\ \mathrm{anterior}\ \mathrm{VI}+\mathrm{thickness}\ \mathrm{of}\ \mathrm{VL}+\mathrm{thickness}\ \mathrm{of}\ \mathrm{lateral}\ \mathrm{VI}\right)/4 $$

EI was assessed at the gray scale level, which was expressed in arbitrary units (a.u.), using ImageJ software. A rectangular region of interest as large as possible was established, excluding the visible fascia and bone in RF from the anterior image and VL from the lateral image. The mean EI inside the region of interest in RF (EI RF) and VL (EI VL) was calculated for each image, and the mean EI from three images for each muscle was used for future analyses. We calculated EI QF using the following equation:

$$ \mathrm{EI}\ \mathrm{QF}=\left(\mathrm{EI}\ \mathrm{RF}+\mathrm{EI}\ \mathrm{VL}\right)/2 $$

The reliability of this methodology was established by Caresio et al. [31], which supported the reliability of our method. We further calculated the interclass correlation coefficient (ICC, 2.1), the standard error of the measurement (SEM), and minimal detectable change (MDC) of EI for 20 randomly selected participants. The ICC was 0.99 for RF and 0.96 for VL (all *P* < 0.01), SEM was 0.73 for RF and 1.86 for VL, and MDC was 7.77 for VL and 8.34 for VL.

### Physical functional tests

The participants performed five functional tests (i.e., sit-ups, supine up, sit-to-stand, 5-m maximal walking and 6-min walk) in a gymnasium. These functional tests were chosen because they have been used in many previous studies as the index of lower limb strength, and the findings of several studies have shown that EI is associated with basic functional capacity and agility [28, 29, 32]. For the sit-up test, the participants lay in a supine position with their knees bent at approximately 80° and their feet flat on the floor. The participants performed as many sit-ups as possible for 30 s with their arms crossed in front of their chest. The examiner held the ankle joints of the participants during the test. The supine up test consisted of measuring the time it took for the participant to go from the supine to standing position as fast as possible using whatever form they preferred. The sit-to-stand test measured the time taken to sit in and stand up from a chair 10 times as quickly as possible, with the participant’s arms crossed in front of their chests. The height of the seat was 40 cm from the floor. For the 5-m maximal walk test, four parallel lines were taped on the floor at 1 m, 6 m, and 7 m (finish line) from the start line (0 m). The participants walked with maximal effort from the start line toward the finish line. The examiner timed between the 1-m and 6-m lines while walking alongside the participant. The 6-min walk test consisted of measuring the distance achieved by walking for 6 min on a 108-m circular course at maximal effort. Markers were placed along the course every 6 m as landmarks, and the examiners counted the laps completed. We verbally encouraged the participants to give their maximal effort. The sit-up, sit-to-stand, and 6-min walk tests were conducted once. The supine up and 5-m maximal walk tests were conducted twice, and the best results were used in the analyses. The ICC values (ICC, 2.1) for the physical function tests indicate that their reliabilities range from “moderate” to “almost perfect” (supine up, 0.85; sit-to-stand, 0.74; 5-m maximal walk, 0.65; 6-min walk, 0.77; *P* < 0.05). The MDC was 1.15 for supine up, 0.34 for sit-to-stand, 0.21 for 5-m maximal walk, and 25.92 for 6-m walk. ICC and MDC were measured in 20 older adults who were recruited from the same community. They were confirmed to match with our participants in age and BMI and were instructed to perform five functional tests two times, following the same procedure, to confirm test-retest reliability.

### Statistical analysis

All values are reported as mean ± standard deviation. Two-way (time × group) analysis of variance with repeated measures over time was used to compare subcutaneous fat thickness, muscle thickness, EI, and physical function parameters. In the case of two-factor interaction of main effects, the Bonferroni post-hoc test was used to identify significant differences. An unpaired Student’s *t*-test was used to compare the variance in the percent change in subcutaneous fat thickness, muscle thickness, and EI between groups. Pearson’s product-moment correlation coefficients were used to determine the associations between the percent changes. The level of significance was set at *P* < 0.05. All statistical analyses were performed using IBM SPSS statistics (version 22.0 J; IBM Japan, Tokyo, Japan).

## Results

Both groups achieved the walking frequency target (W-group: 2.8 ± 1.6 times per week, WR-group: 3.0 ± 2.0 times per week). The WR-group participants performed their home-based resistance training series of exercises with an average of 5.1 ± 2.8 times per week. Participants walked approximately 11,000 steps on their walking training days (W-group: 11,473 ± 2683 steps, WR-group: 11,035 ± 2324 steps). The number of steps taken on non-walking days was significantly lower than that on training days (W-group: 7969 ± 2034 steps, WR-group: 7498 ± 2180 steps). The average numbers of steps taken per day during the 10-week training period were 9117 ± 2360 steps for the W-group and 9306 ± 2417 steps for the WR-group; these values were not significantly different.

There were significant time-by-group interactions for anterior subcutaneous fat thickness, SFT QF, RF thickness, lateral VI thickness, EI VL, and EI QF (Table 1). The EIs of RF, VL, and QF were significantly decreased relative to baseline in both groups after the training intervention (*P* < 0.05). When compared with baseline values, RF thickness significantly increased in the WR-group, whereas the thicknesses of RF and VI-lateral significantly decreased in the W-group after training. Lateral and QF SFT were significantly decreased in the WR-group after training (*P* < 0.05). The percent change from baseline in muscle thickness and EI is also shown in Table 1. There were significant between-group differences in the percent changes in RF thickness, anterior VI thickness, and MT QF. There were also significant between-group differences in the percent changes in EI of the VL and QF.

**Table 1:** Echo intensity, subcutaneous fat thickness and muscle thickness for the walking training group (W-group) and walking and resistance training group (WR-group) in before and after the 10-week training

	W-group			WR-group		
	Before	After	% Change	Before	After	% Change
Echo intensity (a.u.)						
Rectus femoris	76.10 ± 9.28	67.23 ± 7.97^*^	−11.17 ± 8.75	67.98 ± 8.79^†^	57.96 ± 9.96^*†^	−14.69 ± 9.93
Vastus lateralis	63.71 ± 7.53	56.18 ± 8.04^*^	−11.60 ± 9.63	60.07 ± 12.54	44.29 ± 11.17^*†^	−25.74 ± 14.52^†^
Quadriceps femoris	69.90 ± 7.43	61.71 ± 7.04^*^	−11.50 ± 7.26	64.02 ± 9.53^†^	51.12 ± 9.95^*†^	−20.22 ± 9.52^†^
Subcutaneous fat thickness (cm)						
Anterior	0.81 ± 0.22	0.82 ± 0.25	1.49 ± 12.39	0.74 ± 0.28	0.73 ± 0.25	0.91 ± 16.13
Lateral	0.61 ± 0.21	0.63 ± 0.24	4.10 ± 21.36	0.62 ± 0.28	0.52 ± 0.21^*^	−13.04 ± 19.56^†^
Quadriceps femoris	0.71 ± 0.21	0.72 ± 0.23	2.14 ± 12.17	0.68 ± 0.27	0.62 ± 0.23^*^	−6.47 ± 12.43^†^
Muscle thickness (cm)						
Rsctus femoris	1.43 ± 0.31	1.31 ± 0.27^*^	− 7.18 ± 12.25	1.38 ± 0.36	1.46 ± 0.36^*^	7.97 ± 18.50^†^
Vastus intermedius - anterior	1.39 ± 0.42	1.24 ± 0.38	−10.20 ± 13.41	1.45 ± 0.47	1.46 ± 0.46	4.60 ± 25.68^†^
Vastus lateralis	1.63 ± 0.33	1.57 ± 0.32	−2.72 ± 17.68	1.66 ± 0.38	1.72 ± 0.41	6.56 ± 25.33
Vastus intermedius - lateral	1.14 ± 0.37	1.02 ± 0.36^*^	−10.09 ± 21.97	1.48 ± 0.40^†^	1.19 ± 0.30^*†^	−14.34 ± 33.19
Quadriceps femoris	1.40 ± 0.25	1.29 ± 0.24	−7.83 ± 7.70	1.49 ± 0.33	1.46 ± 0.32	− 0.63 ± 16.42^†^

Values were shown as mean ± SD

* *P* < 0.05 vs. Before. ^†^*P* < 0.05 vs. W-Group

After the training intervention, participants in both the W- and WR-groups showed improvement in the sit-up test. The W-group also showed improvements in the sit-to-stand and 5-m maximal walk tests after the intervention (Table 2).

**Table 2:** Functional performance for the walking training group (W-group) and walking and resistance training group (WR-group) in before and after the 10-week training

	W-group		WR-group	
	Before	After	Before	After
Sit-ups (repetitions)	8 ± 6	11 ± 6^*^	8 ± 4	10 ± 5^*^
Supine up (s)	3.2 ± 0.8	3.4 ± 0.6	2.8 ± 0.7	2.9 ± 1.0^†^
Sit-to-stand (s)	13.9 ± 3.0	11.9 ± 3.1^*^	9.8 ± 1.7^†^	10.2 ± 2.1^†^
5-m maximal walk (s)	2.4 ± 0.3	2.3 ± 0.5^*^	2.2 ± 0.2^†^	2.3 ± 0.2
6-min walk (m)	592.6 ± 64.9	620.4 ± 70.4	617.1 ± 51.7	619.0 ± 77.7

Values were shown as mean ± SD

* *P* < 0.05 vs. Before. ^†^*P* < 0.05 vs. W-Group

The correlations of the percent changes in EI QF with SFT QF, MT QF, and physical function are shown in Table 3. We calculated the percent change in the sit-up test using a limited number of participants (W-group, *n* = 22; WR-group, *n* = 26) because some participants scored zero at their baseline assessment. The percent change in EI QF was associated with the percent change in MT QF in both groups. The percent change in EI QF was associated with the percent change in the supine up test in the W-group.

**Table 3:** Correlation coefficient between the percent change of quadriceps femoris echo intensity (EI QF) and that of subcutaneous fat, muscle thickness and physical functions

	W-group		WR-group	
	CC	*P value*	CC	*P value*
Subcutaneous fat in QF	−0.09	0.64	−0.12	0.49
Muscle thickness in QF	−0.46	0.01	−0.35	0.04
Sit-ups	−0.29	0.18	0.10	0.63
Supine up	0.38	0.03	0.18	0.34
Sit-to-stand	0.23	0.22	0.25	0.16
5-m maximal walk	0.13	0.48	−0.17	0.34
6-min walk	−0.36	0.05	−0.06	0.76

*CC* correlation coefficient, *QF* quadriceps femoris

## Discussion

The main findings of this study were as follows: 1) the EI of the thigh muscles significantly decreased in both W- and WR-groups over the study period and 2) changes in EI VL and EI QF in the WR-group were significantly greater than those in the W-group, resulting in significantly lower post-intervention values, which suggests a greater improvement of muscle quality in the WR-group than in the W-group.

According to the American College of Sports Medicine, walking is the recommended endurance exercise for older adults [33]. Therefore, we selected walking as the endurance exercise used in this study and monitored the time the participants spent walking and the number of steps taken. We allowed participants to walk at their own, self-selected pace for several reasons. First, the positive effects of walking have been previously established. Rooks et al. [13] reported that self-paced walking improved physical function parameters, such as balance and stair climbing, and Ryan et al. [16] confirmed that walking reduces IMF. Second, allowing participants to self-select their walking pace reduces the risk of falls and stress fractures. A recent cross-sectional study showed that the risk of falls was greater during both slow and fast walking than during walking at a normal speed [34]. Furthermore, walking at a non-usual speed (slow or fast) can induce excessive fatigue, which increases fall risk in older adults [35]. Therefore, we decided that self-paced walking exercise provided the best balance between stimulating muscle quality improvements and the need for participant safety. The participants took approximately 7500 to 8000 steps per day on non-walking days and 11,000 steps per day on walking days, with an overall average of approximately 9000 steps per day for both groups. Previously, the number of steps taken by healthy and unhealthy older individuals with obesity, peripheral arterial occlusive disease, and claudication or stroke ranged from 3500 to 7000 steps per day [26, 36]. It is speculated that, compared with the subjects of these studies, some of our participants would be active and healthy; in fact, they had no serious health problems, e.g., obesity, endocrine disorder, sarcopenia, and frailty. Through the walking intervention used in our study, our participants increased their number of steps per day by approximately 2500 to 3000 steps; eventually, the overall daily steps had increased by approximately 1.2- to 1.3-fold compared to those in untrained healthy older individuals [37]. This might have contributed to the significant improvements in their functional parameter test results after the intervention. This result is supported by previous reports, which suggest that walking contributes to improvements in mobility and the ability to perform ADL [13]. We measured muscle EI to find the infiltration level of adipose and/or connective tissue [21, 22, 38]. Another striking result of this study was that the EI of the thigh muscles significantly decreased after the 10 weeks walking intervention. This result is similar to the results reported by Ryan et al. [16], who found a reduction in IMF cross-sectional area (as measured by CT) after a walking intervention in obese older women. However, given the different measurement methods (e.g., CT vs. ultrasonography) and the physical characteristics of participants in our study compared with those in the study by Ryan et al. [16], similarities in the results should be interpreted with care. The reduction in muscle use induced by lower limb unloading has been shown to increase IMF in the calf and thigh [39], and Goodpaster et al. [40] reported that increased physical activity prevented an increase in IMF. These results suggest that the amount of activity performed by the lower limb muscles greatly influences their IMF content. We also found a significant decrease in the EI of RF, VL, and QF after the 10-week walking training intervention in our study. This response likely indicates a decrease in IMF content. Improvements in muscle quality may also reduce the risk of type 2 diabetes in older individuals because IMF content has a negative effect on insulin sensitivity [2, 8]. Therefore, walking may be an effective method of improving the quality of the thigh muscles in older individuals and may result in recovery and/or prevention of metabolic syndrome.

We showed a significant decrease in SFT in the WR-group accompanied by a decrease in muscle EI; accordingly, this caused a decrease in SFT and EI in QF (Table 1). In a cross-sectional study, Goodpaster et al. [2] found significant correlation coefficient between subcutaneous fat cross-sectional area and muscle density (*r* = − 0.35, *P* < 0.01), which is the index of fat infiltration level, implying that IMF content was higher if subcutaneous fat area was higher. Therefore, IMF accumulations may change and may be accompanied by a change in subcutaneous fat; however, it is still unknown how regional fat accumulation in the whole body, including subcutaneous, abdominal, intramuscular, intermuscular, liver, and so on, are related to each other. Interestingly, the change was shown only in the lateral region of the WR-group but not in the anterior region (Table 1). This result is inconsistent with that of our previous training experiment [29]. This spot-specific change could be explained by the findings of Akima et al. [28]. They showed that SFT is related with the EI in the lateral (*r* = − 0.40, *P* < 0.05) but not in the anterior region (*r* = − 0.29, *P* > 0.05). However, there are few studies reporting the relationship of longitudinal change in both subcutaneous fat and muscle quality, and the underlying physiological mechanism is still unclear.

The percent changes in the muscle thicknesses of the RF and QF were significantly higher in the WR-group than in the W-group (Table 1). This demonstrates that home-based weight-bearing resistance training effectively increased the muscle size of the participants in our study. This result was partly consistent with the results of previous studies [17, 41] and suggests that home-based resistance training may contribute to preventing sarcopenia and related mobility disorders, falls and fractures, disability, and loss of independence [13, 24, 25]. However, in both groups, some of the muscles examined showed no significant change or even decrease in thickness after the training intervention (Table 1). This was likely because the MT parameter included both skeletal muscle tissue and IMF tissue, even though this parameter was called “muscle thickness” [17, 41]. Thus, if the thickness of the skeletal muscle tissue and/or IMF tissue decreased as a result of the intervention, the measured “muscle thickness” would decrease. EI changes in the W and WR groups suggest that IMF decreased after the 10-week training intervention. However, using ultrasound imaging, it is difficult to determine whether the change in thickness that we observed was due to IMF loss alone or to muscle loss. MRI is considered the gold standard medical imaging modality for analysis of lean and non-lean tissues [3, 4]; however, the problems of cost and accessibility of MRI have been discussed frequently in the literature. For practical reasons, we used ultrasonography in this study. The validity of assessing muscle size on the basis of thickness measurements made using ultrasonography has been shown previously [42], and the validity and reliability of muscle quality measurements made using ultrasonography have also been discussed [20, 23, 31]. Therefore, ultrasonography has been shown to be a suitable imaging technique for studies such as ours and has the advantage of lower cost and greater accessibility than those of MRI.

Our participants took approximately 7500 to 8000 steps per day on non-walking days, and the score of physical functional performance was better than that in our previous reports [19, 29]. These characteristics would imply that participants were very active and did not have obstacle to the exercise and made it possible to achieve our home-based training protocol because participants were required to manage and fulfill their own training quota considering their lifestyle. They actually achieved the target of walking and resistance training frequency. These characteristics of participants would affect the results as well; greater decreases in EI, which indicate greater improvements in muscle quality, were observed after home-based weight-bearing resistance training in conjunction with walking compared with after walking alone (Table 1). Concurrent endurance and resistance training effectively improves both muscle strength and cardiovascular function and has been previously shown to reduce EI by 5% in older individuals [17]. However, this reduction is small compared with the effect on EI after strength training alone shown by Radaelli et al. [41]; they found a 12 to 20% reduction in EI after resistance training of the same duration. Similarly, Akima et al. [3] reported that muscle size inversely determined IMF content. Thus, we hypothesized that the effects on IMF of walking combined with resistance training that induced muscle hypertrophy would be greater than the effects of walking alone. Consistent with our hypothesis, the percent changes in the EI VL and EI QF of the WR-group were significantly greater than those in the W-group (Table 1). However, this result was inconsistent with results reported by Marcus et al. [43]. They confirmed a decrease in IMF cross-sectional area after exercise but failed to find a specific effect of combined training. One reason for this discrepancy may be the characteristics of the participants. IMF content was reported to be affected by many factors, including age, disease status, injury, inactivity, and obesity [44]. Furthermore, race is one of the factors that determine individual difference in IMF content [7]. The different magnitudes of response to the training interventions in our study might also be due to different metabolic responses in the W- and WR-groups. Perhaps, the reductions in IMF that we observed were the result of changes in energy expenditure, fat oxidation, and/or improvements in mitochondrial function [45, 46]; however, we have not determined to what extent these changes occurred in the muscles of our participants.

We found significant correlations between the percent changes in EI QF and the percent changes in MT QF in both groups (Table 3); this result supports the findings of a previous study by Akima et al. [3]. Similarly, Gorgey et al. [47] reported that neuromuscular electrical stimulation of patients with spinal cord injury decreased IMF in conjunction with muscle hypertrophy. Manini et al. [39] showed a significant increase in IMF with muscle loss as a result of lower limb unloading and found that the increased IMF could be statistically explained by muscle loss. Therefore, interventions that increase muscle size, such as resistance training, may more effectively reduce IMF than endurance training interventions. In our participants, not all muscles increased in thickness (Table 1); this was inconsistent with our hypothesis and may suggest that the intensity of the weight-bearing resistance training was not sufficient to induce muscle hypertrophy in all muscles. Many previous studies have used resistance training protocols with increasing loads that require resistance machines or dumbbells [17, 41, 43]; however, lack of access to transportation, unavailability of training programs, labor considerations, and costs are major limitations of these types of interventions. Furthermore, the risk of injury is also greater with this type of training. Considering the balance between risk, simplicity, and versatility, we concluded that home-based weight-bearing resistance training was suitable for our study, and our results suggest that it was effective for improving muscle quality even without causing hypertrophy of all muscles. The lack of evident hypertrophy in some muscles may also be because of the inclusion of IMF in the MT measurement, as discussed previously. A limitation of ultrasound imaging analysis is that it is difficult to precisely differentiate between muscle tissue and IMF.

Our study had several limitations. First, we assigned participants to the W- and WR-groups using a non-randomized controlled procedure. However, we overcame the biases as much as possible by the following procedures: 1) participants were recruited from the same city using the same methods (e.g., public invitation from Nagoya City using public relations magazine and website), 2) participants in each year met the same inclusion criteria (e.g., aged over 65 years, living independently, without serious disease, capable of exercise, and not currently involved in exercise training), and 3) blinding the examiners analyzing the data to the participants’ group. Thus, there were no significant between-group differences in basic parameters such as age, height, weight, BMI, and the number of steps taken on non-walking days. Given these conditions, our participants showed a high level of activity and performances of physical function. Because of these participant characteristics, it would be difficult to apply this training effect to older adults with injury, sarcopenia, frailty, nursing care, etc. Second, we did not have a control group. Previous studies that examined training effects on EI have reported the before and after EI results over a control period; they found that EI did not change over 6 weeks and 12 weeks [17, 48]. Data from a control period may emphasize the effects of the training intervention, but we observed changes in EI that were obviously enhanced by physical activity. Third, we did not measure baseline physical activity level because of restricted participant schedule. However, we analyzed their training logs and calculated the number of steps on a non-training day as the baseline step count. We instructed participants to describe in detail their physical condition, daily steps, and events of daily living comprehensively related with daily activity. Furthermore, we strictly checked them once every 2 weeks through consultation. These managements could contribute to understanding their daily living activity in both training and non-training days.

## Conclusion

Knee extensor muscle EI was significantly reduced after a 10-week walking intervention in older adults and even further reduced by a combined walking with home-based resistance training intervention. Changes in EI were negatively correlated with changes in MT in both groups, suggesting that the mechanical and metabolic stimulation of the trained muscles resulted in EI changes. These results indicate that walking training alone may be useful for improving the muscle quality of older individuals but that it has a lesser overall training effect than walking combined with home-based resistance training. Muscle size and muscle quality were both improved, with concurrent improvements in functional abilities, as a result of the 10-week combined walking and home-based resistance training intervention without the use of conventional resistance training machines.

## Abbreviations

CT: Computed tomography
EI: Echo intensity
IMF: Intramuscular fat
MRI: Magnetic resonance imaging
MT: Muscle thickness
QF: Quadriceps femoris
RF: Rectus femoris
SEM: Standard error of the measurement
SFT: Subcutaneous fat thickness
VI: Vastus intermedius
VL: Vastus lateralis
W-group: Walking group
WR-group: Walking and resistance training group
